# Motor learning mechanisms are not modified by feedback manipulations in a real-world task

**DOI:** 10.1038/s41539-025-00373-8

**Published:** 2025-10-30

**Authors:** Federico Nardi, A. Aldo Faisal, Shlomi Haar

**Affiliations:** 1 UKRI Centre for Doctoral Training in AI for Healthcare, Imperial College London, London, UK; 2 Care Research & Technology Centre, UK Dementia Research Institute, London, UK; 3 Department of Computing, Imperial College London, London, UK; 4 Department of Bioengineering, Imperial College London, London, UK; 5 Chair in Digital Health & Data Science, University of Bayreuth, Bayreuth, Germany; 6 Department of Brain Sciences, Imperial College London, London, UK; 7 School of Psychology, University of Surrey, Guildford, UK

**Keywords:** Learning and memory, Sensorimotor processing, Human behaviour

## Abstract

Error-based and reward-based mechanisms of motor learning co-occur in real-world scenarios but are traditionally isolated in laboratory tasks via feedback manipulations. We examined the distinctiveness of these mechanisms by applying lab-based feedback manipulations to a real-world task. Using Embodied Virtual Reality of pool billiards, we introduced visual perturbations while maintaining full proprioception. 32 participants underwent two sessions, learning visuomotor rotation with either error or reward feedback. The reward-dependent motor variability and inter-trial variability decay - indicators of reward-based learning - were higher in the error-condition. Moreover, post-movement beta rebound (PMBR), a neural marker of learning mechanisms, showed expected decrease under the reward-condition but no consistent trend under the error-condition. These suggest that participants could engage in reward-based learning even without reward feedback. This underscores the complexity of motor learning processes and highlights that visual feedback by itself cannot elucidate the interplay between error-based and reward-based mechanisms in real-world contexts.

## Introduction

Motor learning plays a crucial role throughout our lifespan, shaping human behaviour from a baby learning to walk to a person living with Parkinson’s disease adapting their movements to compensate for tremors. This learning process is considered to be driven by two key mechanisms: *error-based* learning, which relies on quantifiable sensory-prediction errors, and *reward-based* learning, which reinforces successful actions^1^. For instance, when playing billiards, we use *error-based* learning to correct our next shot based on the disparity between the pocket and the actual outcome. Conversely, after pocketing the ball, *reward-based* learning positively reinforces the successful actions, increasing the likelihood of repeating the same movements in the future.

This dichotomy in motor learning mechanisms has been extensively studied in the field of neuroscience. Specifically, *error-based* learning is considered to be based on adaptation mechanisms in the cerebellum^2–4^, while *reward-based* learning is often associated with the brain’s reward pathways in the basal ganglia and the release of neurotransmitters such as dopamine, which play a crucial role in encoding the value of particular motor actions^5–7^.

Understanding the interplay and influence of these error-based and reward-based learning mechanisms is crucial for developing effective strategies to enhance motor learning and skill acquisition^8^. Exploring their interaction in realistic and diverse settings can provide a deeper understanding of how individuals learn and adapt their movements in everyday life^1^.

Typically, these two processes of error-based and reward-based learning are studied as distinct mechanisms in laboratory tasks with few degrees of freedom, isolating these two mechanisms through controlled feedback manipulations^4,9^. However, the unparalleled complexity of human movement cannot be fully captured by such simplistic tasks, which however could be informative of the individual processes that we investigate. Our recent work has shown that in a real-world task of playing billiards, error-based and reward-based learning coexist, with participants using a combination of both mechanisms while predominantly relying on one or the other^10^.

Recent advancements in real-world neuroscience have enabled the study of neurobehavioral processes in natural human behaviour, offering new insights into the complexities of motor learning and skill acquisition^11–14^. However, these approaches come with limitations, as controlling different types of feedback and learning mechanisms is more challenging compared to classical lab-based tasks. To overcome this limitation, we integrated our billiards task into an Embodied Virtual Reality setup. This setup allows for visual manipulations without compromising the complex movements required by a real-world task^15^. The EVR setup synchronises a physical pool table, cue stick, and balls with a virtual environment using optical marker-based motion capture technology. Participants can interact with physical objects simultaneously in both the real and virtual worlds, enabling us to manipulate the visual environment while they engage in realistic movements accompanied by full proprioceptive feedback^16^.

In this study, we leveraged our Embodied Virtual Reality setup to explore the potential of feedback manipulations commonly used in lab-based tasks to differentiate error-based and reward-based learning mechanisms in a real-world motor learning task. Accordingly, we presented the participants with an error-feedback task, were they see the cue ball rolling until the conclusion but than the balls disappear without exposing participants to the reward feedback of pocketing. In a separate session, we presented the participant with a reward-feedback task, where they were shown artificially generated successful trajectories as a form of reinforcement on rewarded trials and were not exposed at all to their real trajectories, receiving no information on their errors. To do so, we employed a dynamic reward zone^9,17^, a commonly used lab-based expedient to isolate reward-based learning while providing a sustained source of feedback to improve.

We hypothesised that by eliminating specific visual information in the EVR setting, participants would exclusively rely on the relevant learning mechanism, resulting in observable differences in their behaviour and neural patterns during the training sessions. In particular, we expect to have several behavioural measures of learning (e.g., inter-trial variability, trial-to-trial change) present in different ways while using the two mechanisms. Similarly, neural trends of Post-Movement Beta Rebound (PMBR) have been related to learning with both mechanisms in diverging manner^18,19^, and therefore can be used to measure how error and reward-based learning are used in motor learning tasks. Our analysis of movement data and adjustments in brain activity following these feedback manipulations provides new insights into the connection between visual feedback and the distinct characteristics of error-based and reward-based learning mechanisms in the context of real-world human behaviour.

## Results

**Fig. 1:**
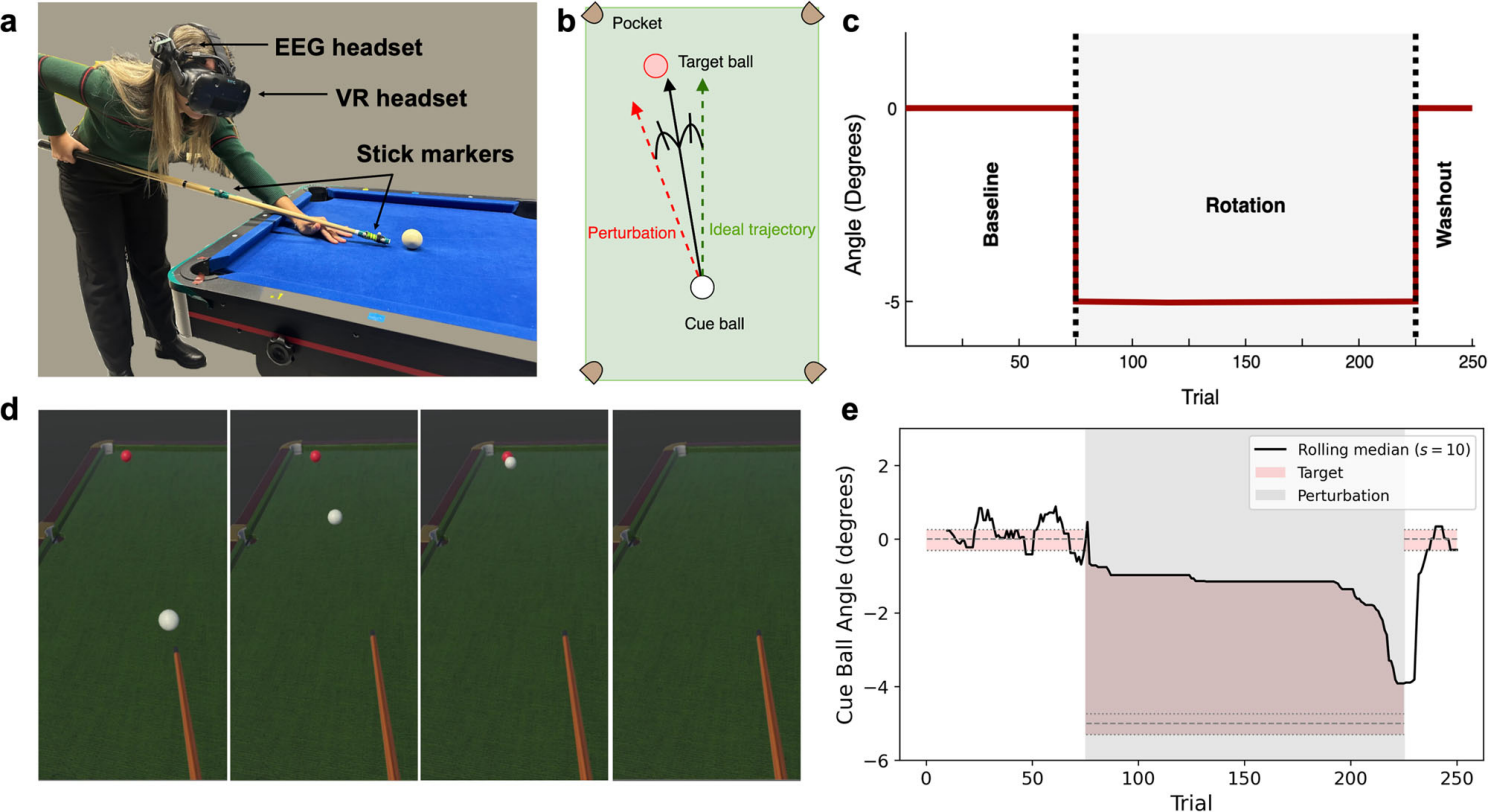
Experimental paradigm. **a** Participant playing in the EVR setup. **b** Perturbation applied during the *Rotation* phase: the trajectory of the cue ball is rotated 5° towards the target pocket side of the table. **c** Structure of the experiment: after 75 trials of full feedback and no perturbation (*Baseline*), a visuomotor rotation is applied on the cue ball trajectory (*Rotation*) for 150 trials; follows a final phase of 25 trials without perturbation and full feedback (*Washout*). The grey area represents the presence of perturbation, whereas the solid red line represents the ideal angle of the cue ball trajectory. **d** Visual feedback for the error task. At collision, the cue ball and the target ball disappear to make the participant correct only based on the trajectory before the hit. **e** Reward zone of an example participant. The grey area represents the presence of perturbation, while the solid black line represents the median of the last 10 rewarded trials. The pink area represents the zone that rewards the participant when the shot is directed in any part of the area: during *baseline* and *washout* it corresponds to the pocketing funnel, while during *rotation* the reward zone is inflated to direct the participant towards the new ideal trajectory. The dotted lines show the range of successful angles, in all the phases of the experiment.

In this study, 32 healthy participants played a pool game with physical objects (table, cue stick, cue ball) while wearing a VR headset and receiving customised visual feedback in the virtual environment (Fig. 1a). In each trial, participants took a single cut shot, aiming to pocket a target ball, placed near the far corner of the pool table, starting from a fixed position. Without any time pressure, the trials ranged in duration from several seconds to over ten seconds, depending on the individual’s pacing (*Med* = 7.34 s, *IQR* = 3.26 s). The experiment included two sessions, 2-days apart, each (Fig. 1c) consisted a *Baseline* phase (75 trials, divided into 3 blocks of 25 trials), a *Rotation* phase (150 trials, 6 blocks) and a final *Washout* block (25 trials).

During the *Rotation* phase, a 5° visuomotor perturbation was applied to the cue ball’s trajectory (Fig. 1b), compelling participants to adjust their aim toward the table’s centre to successfully pocket it. In each of the sessions, during the *Rotation* phase, the participant received either error-only or reward-only feedback through the VR. In the error condition, the balls disappeared after colliding with each other (Fig. 1d), while in the reward condition, shots which were defined successful resulted in an artificially generated successful trajectory (of cue ball hitting the red ball and the red ball pocketing) as a form of reinforcement, and unsuccessful shots received no visual feedback. The rewarded zone (Fig. 1e) for the second condition was defined following widely used reward-based learning experimental paradigms^9,20,21^, using two criteria: (i) shooting within a successful funnel around the pocket, or (ii) when the accuracy of the shot exceeded the median of the previous 10 successful attempts. All participants took part in two sessions in different conditions - one with error and one with reward feedback - shooting towards opposite pockets. The order of conditions and pockets was randomised and counterbalanced across participants.

**Fig. 2:**
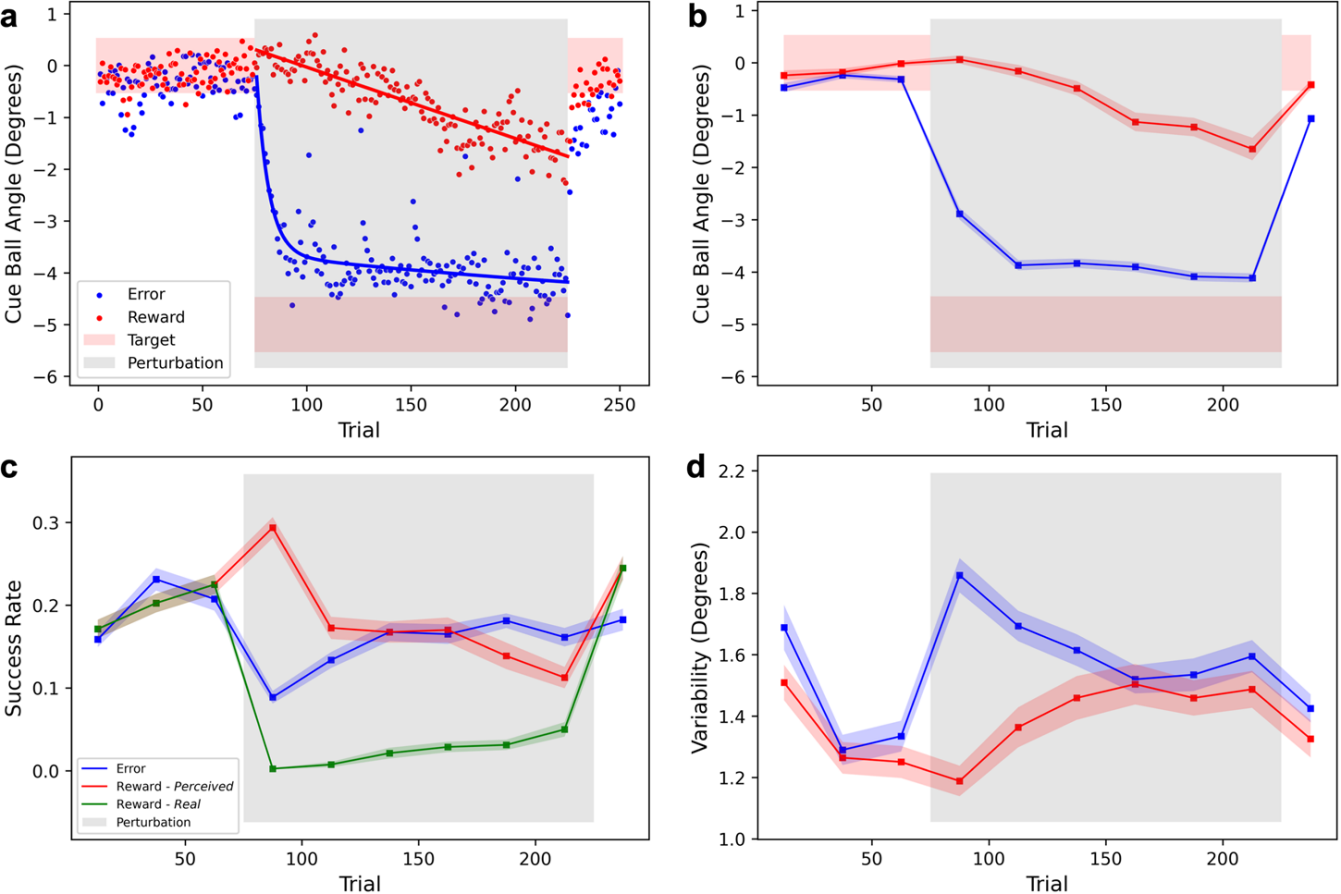
Task performance in error (blue) and reward (red) conditions. **a** Average cue ball trial-by-trial angle over the participants. The pink area indicates the range of successful angles. The bold line is a double exponential for the error data /linear fit for the reward. **b** Average cue ball angle over blocks. **c** Success rate for the *error* condition, vs Perceived (red) and Real (green) success rates for the *reward* condition. **d** Corrected variability, after removal of linear trends. **b**–**d** Each block of 25 trials is represented by its distribution, the means over participants are connected by the bold lines and the shaded areas represent inter-participant variability (standard error of the mean). Grey area represents the presence of perturbation. *N* = 32.

Participants were able to successfully learn and correct for some of the rotation under both error and reward conditions. However, their learning patterns varied significantly due to the feedback manipulation in the EVR setup (see Fig. 2a), confirming our preliminary results^16^.

During the baseline phase, with full visual feedback and no perturbation, participants exhibited similar performance in both conditions. However, during the rotation phase with partial visual feedback and a perturbation on the cue ball’s trajectory, participants learned the rotation more quickly with error-only feedback. This resulted in a larger after-effect during the washout phase. The trial-by-trial directional errors in the error-feedback session showed a fast initial learning phase followed by a sustained slower one, best captured by a double exponential curve (*τ*_fast∣error_ = 6, *τ*_slow∣error_ = 296). In contrast, learning through reward-only feedback progressed at a slower pace, with a linear curve providing the best fit (*b*_0_ = 0.31, *b*_1_ = −0.014).

The block averages displayed a similar pattern (see Fig. 2b), with a significant difference between the two learning trends during the perturbation phase (*F*_mode∣pert_(1, 31) = 73.93, *p* < 0.001; *F*_int∣pert_(5, 155) = 3.55, *p* = 0.027). Despite learning at a slower rate in the reward condition, participants were still able to compensate for at least some of the visuomotor rotation in both conditions.

The slower learning with reward-only feedback can be partially explained by the use of a “reward zone,” which led to an increased perceived success in the early blocks of the rotation. In an error feedback session, a successful shot is simply defined as when the trajectory was heading towards pocketing. With reward-only feedback, there are two types of success: *real* and *perceived*. While real success is equivalent to that in the error feedback condition, where the trajectory was heading towards pocketing, perceived success is based on the received rewards. Early on in rotations, rewards are given whenever the direction is closer to reaching for a rotated pocket than for the original one.

Accordingly, success rates were similar between sessions in the baseline and washout blocks (*F*_mode∣base_(1, 31) < 0.01, *p* = 0.99; *F*_mode∣wash_(1, 31) = 2.32, *p* = 0.13). However, during the perturbation phase we observed very different success profiles (Fig. 2c). The real and perceived success rates during reward-only blocks showed opposite trends (*F*_int∣pert_(5, 155) = 7.27, *p* < 0.001), and the success rates during error-only blocks differed from both perceived (*F*_int∣pert_(5, 155) = 9.57, *p* < 0.001) and real reward (*F*_mode∣pert_(1, 62) = 44.39, *p* < 0.001, *F*_int∣pert_(5, 155) = 1.75, *p* = 0.15).

The variability in shot direction under different conditions (Fig. 2d) closely corresponded with their perceived success rates. To address potential learning trends within a block, we calculated the *corrected variability* by determining the standard deviation of residuals from a block-level linear regression model applied to the cue ball angle data^11^. The participants’ performance exhibited similar patterns during the baseline and washout phases (*F*_mode∣base_(1, 31) = 0.39, *p* = 0.54; *F*_mode∣wash_(1, 31) = 0.41, *p* = 0.53), but demonstrating opposite dynamics during the perturbation phase over time (*F*_int∣pert_(5, 155) = 6.64, *p* < 0.001).

**Fig. 3:**
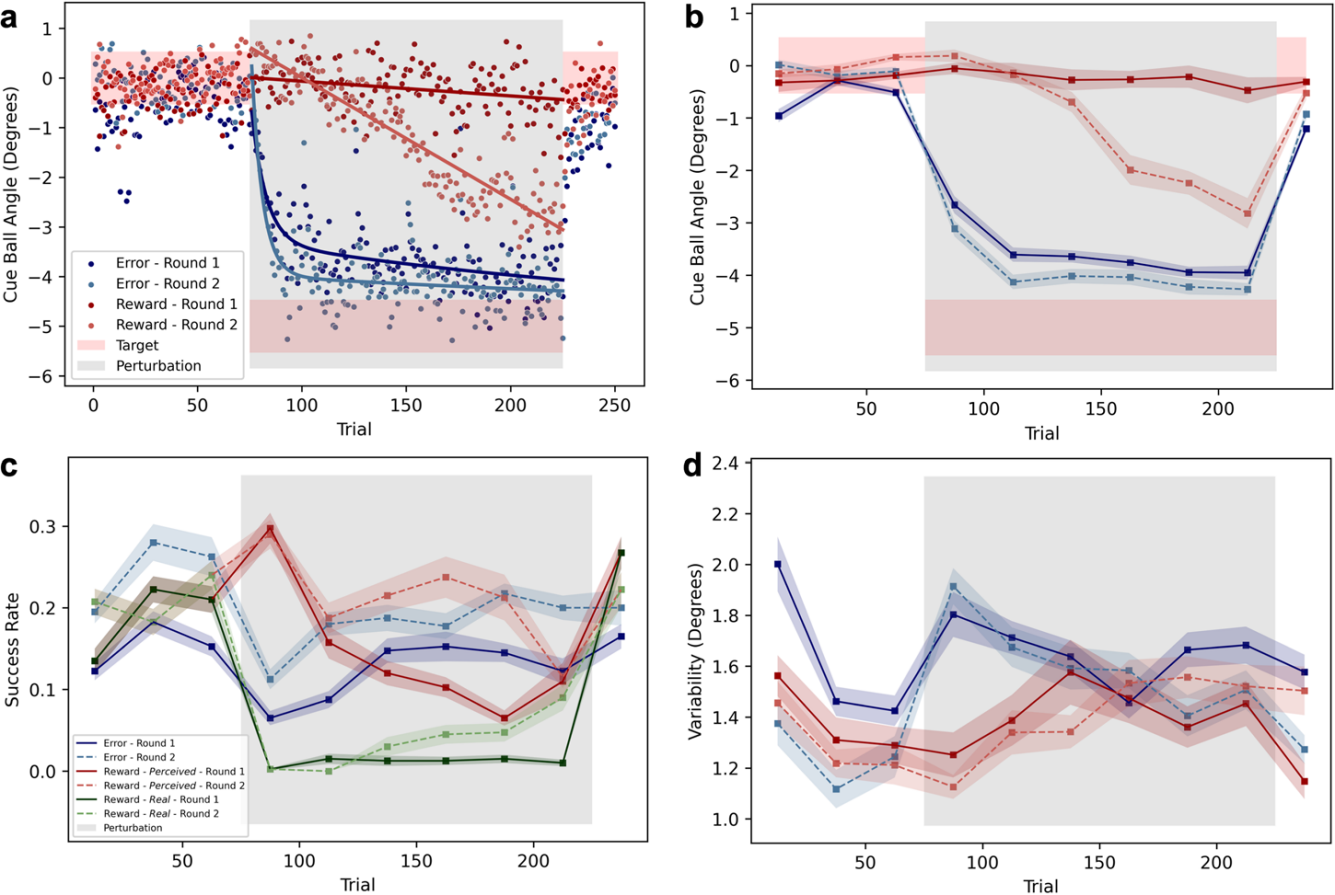
Task performance by sessions: First (dark and solid) vs Second (light and dashed) in error (blue) and reward (red) conditions. **a** Average cue ball trial-by-trial angle. The pink area indicates the range of successful angles. The bold line is a double exponential for the error data /linear fit for the reward. **b** Average cue ball angle over blocks. **c** Success rate for the *error* condition, vs Perceived (red) and Real (green) success rates for the *reward* condition. **d** Corrected variability, after removal of linear trends. **b**–**d** Each block of 25 trials is represented by its distribution, the means over participants are connected by the bold lines and the shaded areas represent inter-participant variability (standard error of the mean). Grey area represents the presence of perturbation. *N* = 32.

Comparing the different sessions within the individual feedback conditions, we observed distinct differences across sessions in the reward condition. The average cue ball angle over time (Fig. 3b) shows a clear distinction between sessions. The participants in Round 1 - who were naive to the task and the EVR - did not learn how to correct for the perturbation, whereas participants in Round 2 - who were less naive to the task and the EVR as they already had a 1st session where they aimed at the other pocket with error-feedback - corrected for most of the perturbation (2.82 ± 0.29 degrees), having results not significantly different from both sessions of the error condition (*W*_*ErrR*1_ = 34, *p* = 0.083; *W*_*ErrR*2_ = 33, *p* = 0.074). The same was found for the success rate (Fig. 3c), which aligns with the learning of the task. Participants who performed the task in their first session rarely experienced real successes once the perturbation was introduced, while those who performed it in their second session (thus, were not completely naive) improved their real success rates over learning, reaching up to 10% of their shots.

The variability of the shots when separating the *First* and *Second* rounds (Fig. 3d) showed similar trends between sessions, unlike the other measured quantities.

### Behavioural metrics suggest mixture in learning mechanisms

To assess the contribution of reward-based reinforcement learning and error-based adaptation in the two feedback conditions,we looked at metrics traditionally linked to reward and error mechanisms.

The first metric we investigated was the decay in inter-trial variability (ITV), which is known to be a distinctive feature of reward-based learning^22–26^. We quantified this by calculating the difference in corrected variability between the first and last blocks of the perturbation phase, as a measure of the uncertainty throughout the learning period.

**Fig. 4:**
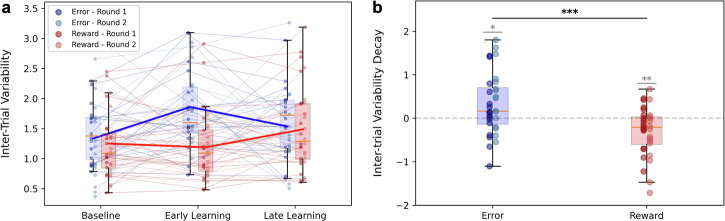
Behavioural differences between error (blue) and reward (red) conditions. **a** Inter-trial variability in the last baseline block (*Baseline* - trials 51–75), first block of rotation block (*Early Learning* - trials 76–100) and last rotation blocks (*Late Learning* - trials 201-225); **b** Inter-trial variability decay from *Early* and *Late* learning. The darker dots represent *Round 1*, while the lighter *Round 2*, for both error and reward. *N* = 32. **a** Solid lines represent the averages considering both rounds. **b** was tested for significant differences against 0 (in grey) and between conditions (in black). *p < 0.05, **p < 0.01, ***p < 0.001.

Surprisingly, while in error-based adaptation we do not expect to see much change in variability, we see an increase in the ITV, sign of exploration, between baseline and early learning (*W* = 289, *p* = 0.003) while it was not significant for the reward (*W* = 542, *p* = 0.69) condition (Fig. 4a). We observed as well a positive decay in variability when providing error feedback (*W* = 142, *p* = 0.02), while the opposite trend was displayed for the reward feedback condition (*W* = 122, *p* < 0.001). Finally, we obtained a significant difference in this metric between the two conditions (*W* = 97, *p* = 0.001, Fig. 4b), whereas no differences were detected during the final baseline block (*W* = 225, *p* = 0.48). Considering the limited learning during the reward condition of the participants who performed it first (Reward - Round 1), we further tested if this condition difference in ITV was driven by the first or the second round. Interestingly, while in the first round neither condition showed more “reward-like” learning (*Round 1*: *U* = 169, *p* = 0.13), in the second round, where the amount of learning was equivalent between conditions, there was a clear condition effect in which the error-feedback showed more “reward-like” learning in terms of ITV decay (*Round 2*: *U* = 211, *p* = 0.003).

**Fig. 5:**
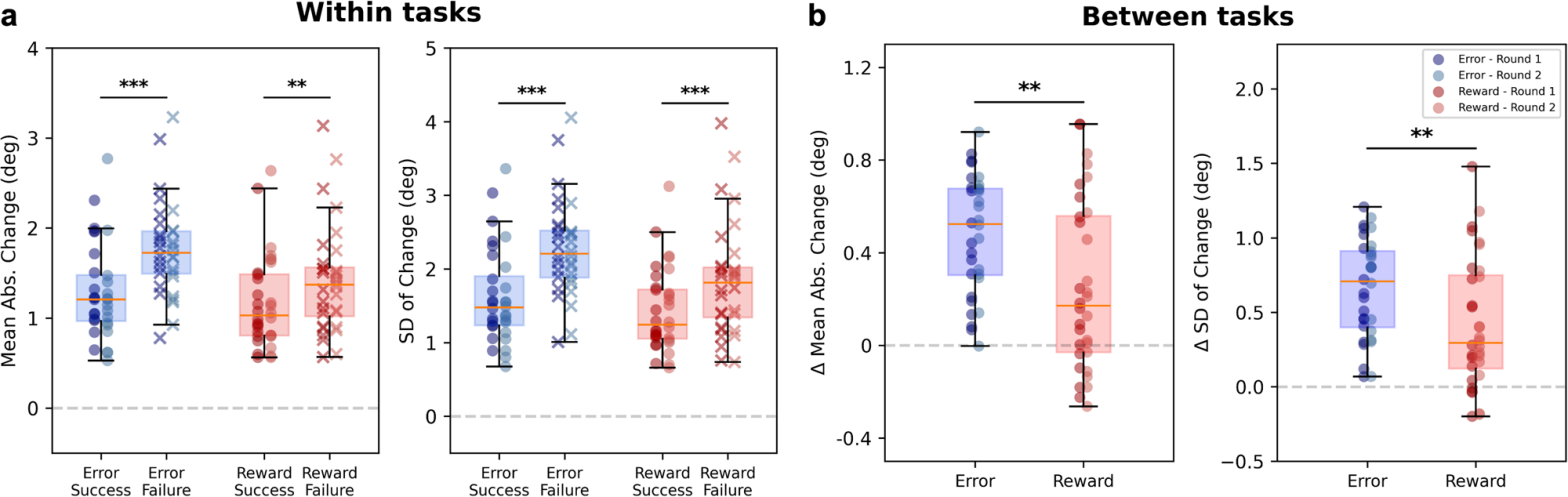
Trial-to-trial angle change after success/failure. **a** Comparison of the average quantities for *error* task - after success (blue dots) and failure (blue crosses) - and *reward* task - after success (red dots) and failure (red crosses). **b** Difference (Δ) between changes after failures and successes for the *error* and *reward* tasks. The Δ is calculated by subtracting the average change after success and after failure trials, for each participant. All quantities are expressed in degrees. The darker dots represent *Round 1*, while the lighter *Round 2*, for both error and reward. *N* = 32. The pairs of panels in (**a**) and (**b**) represent the mean absolute change (left) and the standard deviation of change (right) for the two conditions. Paired tests for significance between success and failure within (**a**) and between (**b**) tasks are reported. *p < 0.05, **p < 0.01, ***p < 0.001.

When examining the reward-dependent motor variability within the error and reward feedback conditions (Fig. 5a), we observed significant differences in the changes of shooting angle between trials following success and failure, for both the error and reward conditions across all the measured quantities. As expected, participants made greater corrections after a failed shot rather than after a successful (rewarded) one (Fig. 5a - first panel), which led to increased exploration when using reinforcement-based learning. This difference was present in both conditions, supporting the hypothesis that reward-based learning was also occurring in the error feedback condition.

Moreover, the comparison between conditions (Fig. 5b) shows significant differences due to the different feedback provided. The error task lead to a greater increase in variability and larger adjustments after failures compared to successes. This suggests that participants were more sensitive to failures in the Error condition, driving more pronounced and consistent motor corrections.

Coherently with ITV, we tested for significant differences between rounds as well in this second measure. For the condition comparison we found here as well that the error condition showed a “reward-like” learning in the second round only, where the learning was comparable, in both mean absolute change (*Round 1*: *U* = 168, *p* = 0.14; *Round 2*: *U* = 189, *p* = 0.02) and standard deviation of change (*Round 1*: *U* = 167, *p* = 0.15; *Round 2*: *U* = 182, *p* = 0.04). On the other hand, while comparing success and failure within conditions, we found significant differences in both conditions in both rounds, and in both ΔMean Abs. Change (*Round 1*: *W*_*Err*_ = 0, *p* < 0.001; *W*_*Rew*_ = 23, *p* = 0.02; *Round 2*: *W*_*Err*_ = 1, *p* < 0.001; *W*_*Rew*_ = 30, *p* = 0.05) and ΔSD of Change (*Round 1*: *W*_*Err*_ = 0, *p* < 0.001; *W*_*Rew*_ = 13, *p* = 0.003; *Round 2*: *W*_*Err*_ = 0, *p* < 0.001; *W*_*Rew*_ = 7, *p* = 0.001).

### EEG neural metrics suggest mixture in learning mechanisms

**Fig. 6:**
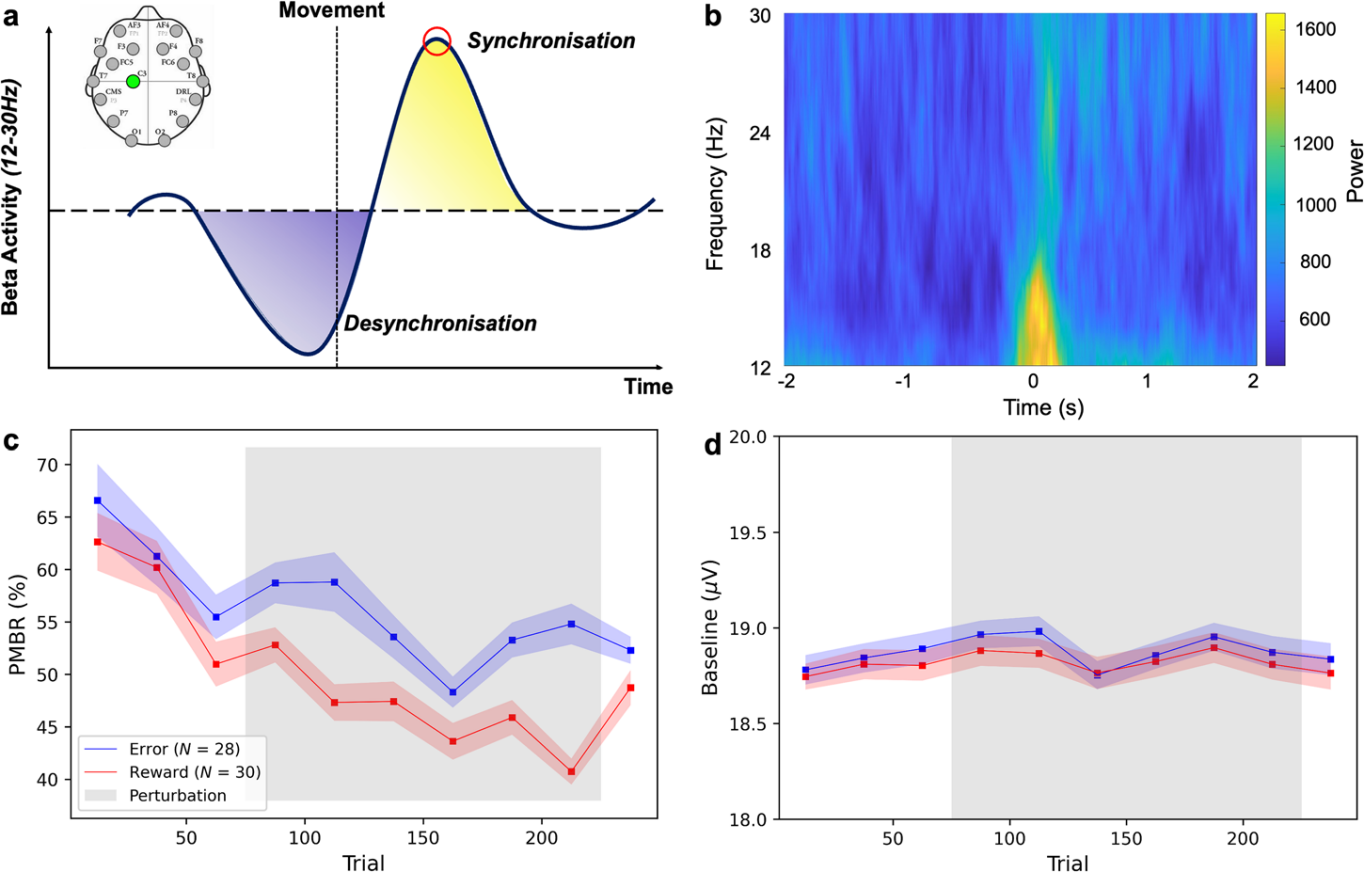
Post-Movement Beta Rebound. **a** Graphical representation of *β*-band *Event-Related Desynchronisation* (in blue) and *Event-Related Synchronisation* (in yellow). Circled in red is the *Post-Movement Beta Rebound* peak considered for the analysis. In the upper left angle, the *Emotiv* EPOC+ electrode map with C3 in green, the channel used for the PMBR derivation; **b** Time-frequency plot for an example session; **c** Average *Post-Movement Beta Rebound*; **d** Average baseline power for the PMBR calculation. **c**–**d** All quantities are calculated within blocks of 25 trials over *error-based* condition (blue) and *reward-based* condition (red). The shaded areas represent the variability between the participants (standard error of the mean) and the grey area represents the presence of perturbation.

During the experiments, we monitored the players’ brain activity to examine trends in Post-Movement Beta Rebound (PMBR) throughout their learning process. PMBR is defined as the peak in beta activity following movement cessation (see Fig. 6a) and is a well-established marker of sensorimotor cortex activation. It is associated with motor learning, and its trend is expected to depend on the underlying learning mechanism^10,18,27–29^.

The increase in beta power was evident in the data (see example in Fig. 6b) for all individual sessions. Both error and reward conditions exhibited similar PMBR trends during the baseline, with a consistent decrease over time. However, a distinct difference emerged in the PMBR trend during the rotation phase (Fig. 6c). The reward sessions showed a continuous decline (*b*_block_ = −1.955, *p* < 0.001, 95% CI: [−2.863, −1.047]), while participants in the error condition did not exhibit a significant trend (*b*_block_ = −1.185, *p* = 0.09, 95% CI: [-2.534, 0.164]). During the washout, the average PMBR remained relatively consistent between conditions without significant differences (*F*_mode_(1, 56) = 0.69, *p* = 0.41). No significant baseline differences were identified between feedback types (*F*_mode_(1, 56) = 0.07, *p* = 0.79) or trends linked to the experimental phase (*F*_int_(9, 504) = 0.24, *p* = 0.99), ensuring that changes in PMBR were not driven by underlying shifts (Fig. 6d).

When the data was split according to sessions, consistent with the behavioural measures, we observed the same trends as in the two-conditions split. The error condition showed a non-significant trend, even though the magnitude differed between sessions (*b*_block∣first_ = −2.019, *p* = 0.11, 95% CI: [-4.495, 0.458]; *b*_block∣second_ = −0.462, *p* = 0.49, 95% CI: [−1.775, 0.851]). In contrast, the reward condition displayed a significant decreasing trend over the learning process in both rounds (*b*_block∣first_ = −2.405, *p* < 0.001, 95% CI: [−3.629 −1.180]; *b*_block∣second_ = −1.504, *p* = 0.03, 95% CI: [−2.846, −0.163]).

## Discussion

In this study, we explored the impact of feedback manipulations on motor learning and the use of different learning mechanisms in a real-world task. By employing an Embodied Virtual Reality setup, we introduced a visuomotor perturbation and manipulated the feedback provided to participants during this real-world task. Our findings indicate that using either error-only or reward-only visual feedback was insufficient to induce learning solely based on error-based adaptation or reward-based skill acquisition, respectively. Both behavioural and neural data suggest that even when the reward feedback (ball pocketing) was removed in the error-only condition, participants still engaged in “reward-like” learning during this task. It seems that participants found sufficient rewards in observing what appeared to be the correct ball trajectory, similar to how professional basketball players celebrate successful shots before confirming if the ball goes into the net.

The results demonstrated a clear difference in how individuals adapted to the visuomotor perturbation when receiving error-only versus reward-only feedback. Participants adapted more rapidly to the cue ball rotation in the error feedback condition compared to the reward feedback condition, in accordance with previous research suggesting that adaptation to errors occurs faster than adaptation based on rewards^30,31^. Furthermore, the difference in learning was even more pronounced between sessions. Participants who experienced the reward condition first showed no learning, whereas those who had previously completed the error condition were able to correct for most of the perturbation.

The definition of the reward zone played a key role here. The increase in received reward during the early rotation phase of the reward-only feedback condition did not encourage exploration and learning, as evidenced by the reduced inter-trial variability in those blocks. The dynamic reward zone, which decreased over time, made the task more challenging, leading to a decrease in perceived success and an increase in variability and exploration over the rotation blocks of the reward-only condition.

The dynamic reward provision was designed to guide participants on different paths without excessive or insufficient rewards, following established paradigms in lab-based motor learning tasks^9^. However, due to this dynamic nature, participants did not experience an abrupt perturbation and instead noticed the changes slowly, which may have affected their perception of the rotated trajectory.

To overcome this limitation, future research might involve the use of a paradigm that involves a gradual visuomotor rotation applied on the trajectory of the cue ball, instead of an abrupt change, so to balance the learning in the two conditions.

As a result, in the reward-only feedback condition, the reduction in directional error exhibited a slow and linear learning curve. Similarly, the *real* success rate in the reward condition showed a gradual, linear improvement mirroring the decline in directional errors. In contrast, the error-only feedback condition displayed a learning curve for the decrease in directional error that followed a double exponential pattern, consistent with typical adaptation tasks [e.g., ref. ^32^] as well as performance in the real-world billiard task^11^. While feedback manipulations of error and reward are often thought to induce distinct learning mechanisms in lab-based tasks, our results suggest a more complex picture in this real-world setting.

The decay in inter-trial variability, a measure traditionally associated with skill learning, revealed intriguing differences between the error and reward conditions^22–24,33^. In the error condition, we observed a decline in inter-trial variability from the beginning to the end of the perturbation phase. Conversely, the reward condition displayed a more fluctuating and diverse pattern of inter-trial variability, without a clear decay trend across participants. This suggests a continuous exploration of different movement strategies, which aligns with the directional error and success rates in the reward-based feedback condition, indicating that participants were still learning by the end of the rotation phase.

Finally, the increased exploration after a failure in both conditions showed how “reward-like” motor learning was used to learn the correct movements in both conditions.

Overall, these results converge to the idea that participants did not exclusively rely on the learning mechanism related to the specific feedback provided, especially in the error condition, where there are clear signatures of “reward-like” learning.

To further validate the differences in learning measures between real-world and lab-based tasks, we also examined the neural activity of participants, specifically the Post-Movement Beta Rebound (PMBR) - the increase in power of neural beta oscillations (12–30Hz) at the end of movement.

PMBR is known to increase over learning in error-based adaptation tasks^18,27,28^, whereas in reward-based learning tasks, PMBR is expected to decrease (itself or its magnetic resonance spectroscopy correlate) over the learning^29,34,35^. This divergence has been attributed to the projections of GABA activity for error- and reward-based learning from the cerebellum and basal ganglia, respectively^36,37^.

Our results indicate that participants initially exhibited a high PMBR, which then decreased during the baseline sessions, as is typical of reward-based learning tasks (e.g., ref. ^29^). This was expected, as participants were learning the EVR environment and the pool task. During the rotation phase, after the introduction of perturbation and partial feedback manipulation, the overall PMBR continued to decay for both feedback conditions. In the reward-only condition, there was a clear decay in PMBR, which is expected in a reward learning task without error-based adaptation. For the error-only condition, the PMBR trend was mixed, with a decay over the early learning phase suggesting the use of reward-based reinforcement learning. However, the PMBR increased over the later part of the rotation phase, where the errors likely became too small to reliably predict reward from the ball trajectory, leading to a shift towards implicit error-based adaptation. This implicit component is evident in the aftereffects observed during the washout phase.

Our findings suggest that participants engaged in a combined use of error- and reward-based learning in the real-world scenario, even when error or reward feedback was withdrawn. Both the behavioural and neural results indicate an interaction between these mechanisms, despite the attempts to isolate them through feedback manipulations. The richness of stimuli and experience in the real-world task appears to have limited the effects of these feedback manipulations. This is particularly evident in the error-only condition, where the participants likely had enough information from the cue ball trajectory to preserve their success and utilise reward-based reinforcement. Similarly, in the reward-only condition, while we withdrew visual sensory-error feedback, the participants still experienced full proprioceptive feedback, which could have guided some error-based adaptation.

While our study provides valuable insights into the interaction between error- and reward-based learning mechanisms in a real-world scenario, it is important to acknowledge certain limitations that may have influenced the findings. First, the sample size of participants could impact the generalisability of the results, and a larger and more diverse participant pool could offer a broader perspective on the dynamics of error- and reward-based learning in motor tasks.

Secondly, participants completed two experimental sessions spaced at least 48 h apart. Each condition was performed on a different target pocket, requiring a physical reorientation and rotation to the opposite side of the table. This design was intended to reduce potential carry-over effects between sessions. Nonetheless, some degree of generalisation from the first to the second session cannot be entirely ruled out. While the change in direction and the temporal gap likely mitigated direct transfer of learned strategies, the familiarity gained during the first session—particularly in terms of task structure and spatial exploration—may have influenced subsequent performance. Thus, we acknowledge that prior exposure may have conferred a general task advantage beyond the specific condition.

Third, the consideration of PMBR as a neural marker for the learning mechanisms may not have fully captured the complex neural processes associated with error- and reward-based learning. Future studies could explore a more comprehensive assessment of neural activity across different brain regions to provide a deeper understanding of the underlying mechanisms and identify patterns beyond PMBR. Fourth, the way the reward feedback was provided did not allow participants to fully correct the perturbation, due to the limited information available. Different methods to provide optimal reward information need further exploration, as the current reward zone method may be sufficient for lab-based tasks but could not be sufficiently informative in real-world scenarios. Finally, while the use of EVR provided a valuable context for studying error- and reward-based learning in real-world situations, it also introduced additional variables and complexities, such as the use of Virtual Reality, which may have influenced the observed dynamics.

Lastly, while the reward-dependent motor variability is considered to be a marker of reward-based learning, since failures would occur on trials with larger errors, participants could make larger changes in response to the error, and we cannot prove it was in response to a reinforcement signal per se.

The findings from this study challenge the notion that learning mechanisms can be isolated solely through feedback manipulation. The observed differences in variability decay, reward-dependent motor variability, and neural activity between error-only and reward-only tasks underscore the intricate nature of motor learning and adaptation in dynamic environments. While feedback manipulation shapes the learning process and influences motor performance, it is clear that participants do not rely exclusively on single learning mechanisms, even when receiving partial visual feedback. Instead, they engage in a holistic learning process that incorporates various environmental cues and internal computations.

In conclusion, this study provides valuable insights into the complexities of human motor learning and performance, emphasising the need for a nuanced understanding of the interplay between error-based and reward-based learning mechanisms. The findings have important implications for developing more effective learning interventions and rehabilitation strategies. Further research in this area holds the potential to advance our knowledge of motor learning processes and inform the design of interventions that better align with the inherent complexities of human motor adaptation.

## Methods

### Experimental setup

Our experimental setup combines a physical pool table with Embodied Virtual Reality to accurately measure and adjust participants’ actions. Participants play an actual pool game while wearing an HTC Vive headset and receive customised visual feedback in the virtual environment, tailored to the targeted learning mechanism (Fig. 1a). The physical cue stick used in the game is faithfully replicated in VR, with its geometric characteristics and marker positions streamed through 4 high-precision Optitrack cameras (accuracy of ± 0.2mm), enabling participants to perceive it as a virtual object.

This embodiment allows participants to physically interact with real-world objects, experiencing complete somatosensory and proprioceptive feedback. Participants shoot a physical cue ball, maintaining haptic feedback and realistic collision sounds. However, artificial sounds represent collisions between the cue ball and target ball, which are solely virtual but programmed to adhere to real physics like other objects in the virtual environment (for validation and further environment details see^11,15^). Pre-experiment calibration using cameras and VR controllers attached to the pool table ensures alignment between the dimensions of the virtual and real tables, enhancing the realism.

The Embodied Virtual Reality paradigm was developed using Unity software programmed in C#. Additionally, the setup integrates a 14-channel wireless electroencephalogram device (eMotiv EPOC+) to record brain activity during the experiments. This device can be easily worn alongside the VR headset without interference.

### Experimental design

32 healthy volunteers (20 men and 12 women) aged 20 to 25 with normal or corrected-to-normal vision and little to no experience in playing pool, participated in an experiment. The experiment included two sessions which took place on different days, 24 to 48 h apart. Each session (Fig. 1c) consisted of a *Baseline* phase (75 trials, divided into 3 blocks of 25 trials), a *Rotation* phase (150 trials, comprised of 6 blocks of 25 trials) and a final *Washout* block (25 trials). In each trial, participants took a single cut shot, aiming to pocket a target ball placed near the far-left or far-right corner of the pool table, starting from a fixed position, and with no any time pressure. The condition order and pocket selection were randomised and counterbalanced between and within participants, who all took part in two sessions in different conditions - one with error and one with reward feedback - shooting towards opposite pockets.

During the *Rotation* phase, a 5° visuomotor perturbation was applied to the cue ball’s trajectory (Fig. 1b), compelling participants to adjust their aim toward the table’s centre to successfully pocket it. Specific feedback related to the condition was provided through virtual reality during this rotation phase. The task was structured to encourage participants to rely on a particular learning mechanism by concealing visual information that contributed to the opposite learning mechanism.

In the error-based feedback condition, the ball trajectories were hidden after colliding with each other, forcing participants to rely solely on cue ball trajectory errors for progression, as they could not see the target ball being pocketed. Conversely, in the reward feedback condition, the balls were hidden after the cue ball was shot. Successful shots resulted in participants being shown an artificially generated successful trajectory (of cue ball hitting the red ball) as a form of reinforcement, according to widely used reward-based learning experimental paradigms^9,20,21^. In case of unsuccessful shots, no cue ball trajectory was displayed.

The region where a reward was provided was established using two specific criteria: (i) shooting within a successful funnel around the pocket, or (ii) when the accuracy of the shot exceeded the median of the previous 10 successful attempts. The defined successful trials assisted participants in making necessary adjustments without error feedback.

It is important to note that learning in the reward-based condition is naturally slower due to the type of feedback provided. Error-based feedback gives more detailed information, helping participants understand how much they need to adjust—whether the correction should be small or large. In contrast, reward-based feedback only tells them if they succeeded or not, without indicating how close they were. Additionally, the reward zone is designed to give positive feedback even for small corrections in the right direction, which can reduce the need to explore or improve further. As a result, learning is expected to progress more slowly in this condition.

A previous pilot study aimed to determine the optimal level of reinforcement^38^. Providing too many rewards would allow participants to quickly reach the correct path, but may reduce their interest. Conversely, setting the reward threshold too high would make it difficult for participants to adjust in a reasonable time due to a lack of guidance. To verify this, several participants took part in pilot experiments where trials were considered “successful” if they exceeded a specific percentage of earlier rewarded trials. The optimum threshold was found by surpassing both the 40^*th*^ percentile and median performance levels, which were close; therefore, we prioritised meeting the median requirement for easier explanation without significant compromise.

All experimental procedures were approved by the Imperial College Research Ethics Committee and performed in accordance with the Declaration of Helsinki, and all subjects gave their informed consent before participating in the study.

### Behavioural data analysis

The data were pre-processed to address extreme values. Specifically, a threshold was established to remove outliers in terms of cue ball directional errors during the baseline (*μ* ± 3*σ*), and it was applied as well in the perturbation learning zone. This procedure removed 1.79% and 3.27% of the data for error-based and reward-based conditions, respectively.

To enhance the robustness and reliability of the observed trends, all primary results were analysed using blocks of 25 trials. As no significant differences were found between shots aimed at the left versus right pockets, data from both directions were combined to increase statistical power. However, differences did emerge between participants depending on whether they performed the task in their first or second session with the same feedback type. Consequently, when relevant, analyses were conducted separately for each session. Participants were initially divided into four groups based on the task performed in their first session and the specific target pocket — *error-left*, *error-right*, *reward-left*, and *reward-right* — ensuring both condition order and shot direction were counterbalanced. Since a condition-order effect was present but no pocket-direction effect was detected, we aggregated groups based on condition order, and all results are reported by session round.

The primary focus of the behavioural data was on the directional error made by the participants in each shot. After defining the ideal successful trajectory (set as 0°), the angle of the cue ball was measured by subtracting that value and analysing the deviations in the direction of the shot^38^.

The trial-by-trial directional error was used to measure learning. When the visuomotor rotation was introduced during the rotation phase, the “ideal” angle changed from 0° to -5° due to the rotation, and all results are presented accordingly (Fig. 1c). To determine the best fit between double exponential (full learning) and linear (partial learning) for the learning curves of the two feedback conditions, we performed an F-test comparing the residual sum of squares of the two curves. The parameters of the curves are reported in the results and referred to as *τ*_fast_ and *τ*_slow_, respectively, for the fast and slow components of the exponential curve. The linear fit is reported through *b*_0_ and *b*_1_, representing respectively the intercept and slope of the linear model.

Another aspect of the game that was considered was the success rate, which had two distinct components: the *real* success rate and the *perceived* success rate. During the baseline and washout phases, when no changes were introduced, these two rates were aligned. In the perturbation phase of the error feedback condition, the latter was missing because participants lacked visual information about the success of their shots. Conversely, in the reward feedback session, the two rates were both present but differed significantly, as the perceived success was based on the rewards given according to the reward zone, while the real success referred to the actual instances when the target ball was pocketed.

To account for uncertainty in the shots and maintain consistency with previous research, the variability of the shots was analysed. Specifically, the focus was on measuring the corrected variability, which involved calculating the standard deviation of the residuals after removing any linear trend from the directional error in shots at a block level. This approach remained valid even when an overall exponential trend was present at the session level, as a sufficiently linear trend was observed within blocks of 25 trials.

The methodology involved the use of repeated-measures and mixed ANOVA, as well as mixed-effect linear models to analyse the repeated measurements from each participant. The selection of repeated-measures was made when all participants had multiple measurements for both sessions, whereas the mixed ANOVA was selected in case of differences between conditions. Non-parametric tests, such as paired Wilcoxon Signed-Rank tests and Mann-Whitney tests, were utilised for sample testing and comparisons to account for non-normal distributions of the samples. Specifically, Wilcoxon Signed-Rank tests were employed to compare single samples against a 0 median or paired samples, while Mann-Whitney tests were used to compare the different samples.

The ANOVA tests reported in the results sections refer to the baseline (*F*_•∣base_), perturbation (*F*_•∣pert_), or washout phases (*F*_•∣wash_), and were executed between different modes (*F*_mode∣•_) or the interaction between mode and time (*F*_int∣•_).

### Behavioural measures of learning

To further validate the behavioural results obtained with the two individual mechanisms, two well-known measures of motor learning were used to demonstrate differences between the feedback: inter-trial variability and reward-dependent motor variability.

Inter-trial variability (ITV) is commonly used as an indicator of motor skill learning, with reductions in variability over time reflecting increased consistency and refinement of movement^22–24^. It was assessed at the block level, considering the corrected variability across the 25 trials within each block. We assessed the decay of ITV by comparing the baseline (last block - trials 51 to 75), early learning (first block - 76 to 100), and late learning phases (last block - 201 to 225).

The reward-dependent motor variability is derived by comparing the trial-to-trial changes in performance after a success or a failure. This measure is traditionally linked to reinforcement-based motor learning and has been shown to increase after unrewarded trials, reflecting the system’s effort to explore new motor strategies to find success^39^. The reward-dependent motor variability was calculated considering the distributions of changes in shot angle after successful and unsuccessful trials for each participant.

### EEG acquisition and pre-processing

The EEG data was collected using an Emotiv EPOC+ headset, a wireless 14-channel system positioned under the VR headset. The data was sampled at 256Hz and aligned with the VR data through the trial timestamps. The entire pre-processing pipeline was executed in MATLAB using the EEGlab^40^ and Fieldtrip^41^ toolboxes.

After applying high-pass and low-pass filtering with a FIR filter at 1Hz and 45Hz, respectively, the data was cleaned using the EEGlab *clean_rawdata* function. This function automatically removes artifacts from the signals, including bad portions of data and problematic channels, using the *Artifact Subspace Reconstruction* algorithm^42^. Specifically, it identifies channels with a large amount of noise based on their standard deviation and poorly correlated channels. To retain as much informative data as possible, the default rejection threshold for channel correlation was gradually decreased by steps of 0.05 until no more than three channels were removed or the minimum correlation reached 0.6. This approach aims to minimise the loss of informative data while preventing excessive removal of channels or retention of non-informative data.

Furthermore, the signal windows were removed based on thresholds for the maximum standard deviation of bursts and the maximum fraction of contaminated channels permitted in the final output data for each considered window. The specific parameters were selected through a manual examination of the sessions’ time-frequency plots, evaluating 4 combinations of parameter settings with increasingly strict criteria.

We extracted epochs from the EEG data centred on the onset of cue ball movement, which provided a reliable temporal reference for the participants’ actions. This approach aligns with prior evaluations of Event-Related Potentials (ERP) and event-related spectral perturbations (ERSP), including the work of ref. ^10^. In motor neuroscience, a key neural signature observed after voluntary movement is the Post-Movement Beta Rebound (PMBR)—a transient and prominent increase in beta oscillation power (13–30 Hz) across the sensorimotor network, typically following movement cessation^43^. PMBR has been associated with motor learning and is thought to reflect GABAergic activity, with its amplitude modulated differently across learning mechanisms—rising during error-based adaptation and decreasing during reward-based learning^18,28,29,35^. In our study, epochs were set to 9 seconds in duration around each billiard shot to capture baseline and post-movement activity, while allowing sufficient margins to observe fluctuations in beta power. However, as PMBR is typically detectable within 1000 ms after movement offset^19^, we focused our analysis on this critical window to identify peak beta activity and quantify PMBR dynamics.

After the epoch extraction, we performed an *Independent Component Analysis* on the data to remove muscle-, eye-, and heart-induced artifacts. Afterwards, we interpolated the C3 channel from the other existing channels using spherical splines and a topological map consistent with the international 10-20 EEG system, which positions C3 over the left motor cortex^44^. This channel placement was informed by previous studies on motor learning^18,27^ and the right-handedness of all participants.

### EEG time-frequency analysis

After preprocessing the EEG data, we conducted a time-frequency analysis to investigate the brain activity associated with the participants’ movements during the experiment (12–30Hz - *Beta* band), while providing individual feedback. The time-frequency domain transformation was performed on each block using a convolution with complex Morlet wavelets in 0.8 Hz steps and 3 millisecond cycles.

To ensure comparability between various sessions within and between participants, we standardised the data so that all sessions had equal total power in the specific frequency range. Specifically, we calculated the total power within the 8–45 Hz range to determine the normalisation factors for each separate session^45^. This normalisation method guarantees that variations in beta are more noticeable and accurate, allowing for precise detection of fluctuations that are solely due to the signal itself, as potential trends and patterns would be observable across all frequencies and not just in the beta band. Furthermore, normalising across sessions eliminates any trends in the baseline, which we define as the median power over each session, as the correction is also applied between the different blocks of the same session.

The subsequent analysis involved calculating the EEG power change in response to events as a percentage change, normalised relative to the median power evaluated in 1Hz steps over each block. This approach was necessary due to the absence of a clear baseline during the task^18,27,28^. The process involved subtracting one from the normalised value and then multiplying by 100 to obtain a comparable percentage scale measure. To ensure robustness, we calculated the peak value on the average signal across the beta-band frequencies and derived one value per trial. Then, we took the median of these peak values across various trials to obtain a PMBR value for each block.

Additionally, to ensure data reliability, we established criteria to include or exclude specific sessions in the conditions analysis. EEG data can have missing values, possibly due to electrode misplacement as the task required significant movements. Blocks of an individual session were included in the EEG time-frequency analysis only if at least half of the block (13 trials) had available data. Entire sessions were removed if at least 3 blocks were missing, to accurately capture trends and avoid including noisy information.

After selecting reliable sessions, missing data were imputed separately for error and reward sessions, using *Probabilistic Principal Component Analysis* (PPCA). This method samples from the conditional distribution of missing values given the observed data^46^. For error and reward condition, respectively, 4% and 6% of the PMBR values were imputed, always subject to the strict threshold of having at least 8 blocks already present, per session. The overall data pre-processing reduced the number of participants in the EEG analysis from 32 to 28 and 30, respectively for the error and reward conditions. Finally, all block values were aggregated by block and reported by condition.

## Data Availability

Data is available on FigShare: 10.6084/m9.figshare.30343489.

## Code availability

The code for reproducing the results from the shared dataset is also available on FigShare. All the analyses and figures were derived in Python 3.
